# SCR-Net: A novel lightweight aquatic biological detection network

**DOI:** 10.1371/journal.pone.0324067

**Published:** 2025-06-09

**Authors:** Tao Li, Yijin Gang, Sumin Li, Yizi Shang

**Affiliations:** 1 School of Human Settlements and Civil Engineering, Xi’an Jiaotong University, Xi’an, China; 2 School of Information Engineering, Minzu University of China, Beijing, China; 3 State Key Laboratory of Simulation and Regulation of Water Cycles in River Basins, China Institute of Water Resources and Hydropower Research, Beijing, China; 4 Department of Engineering, University of Cambridge, Cambridge, United Kingdom; Thapar Institute of Engineering and Technology: Thapar Institute of Engineering and Technology (Deemed to be University), INDIA

## Abstract

Marine biological detection is critical to environmental conservation and the use of marine resources. In actual applications, detecting aquatic species quickly and accurately while using few resources remains a difficulty. To address this problem, this research proposes a novel fast and efficient lightweight target detection network (SCR-Net). First, a fast and lightweight Spatial Pyramid Pool ELAN (SPPE) module is proposed and implemented, which enhances the model’s performance by leveraging ELAN’s effective feature aggregation ability and SPPF’s spatial pyramid pooling capacity. Second, a cross-scale feature fusion pyramid (CFFP) structure is introduced, which significantly reduces the number of parameters and computational cost during feature fusion. Third, a lightweight feature extraction module named RGE is designed, utilizing low-cost processes to create duplicate feature maps and reparameterization to drastically accelerate model inference. Compared to the baseline model, SCR-Net has 57.4% fewer parameters, 37% less computation, and an mAP@0.5 of 83.2% on the DUO dataset. Ablation experiments validate the effectiveness of the proposed modules, and comparative experiments on DUO and UDD datasets demonstrate that SCR-Net achieves superior overall performance compared to existing lightweight state-of-the-art underwater target detection models.

## 1 Introduction

The ocean is an essential component of the planet, containing many biological resources and having a significant influence on human growth and advancement. Monitoring marine species is an essential aspect of maintaining marine ecosystems and protecting marine biodiversity. Target detection, as an essential part of computer vision, has been widely employed in underwater target detection technologies [1,2]. However, large-scale exploration equipment and human resources are still the mainstays of target identification in underwater environments today [3]. This makes underwater detection results easily influenced by human variables, resulting in a significant inaccuracy in the final result. Additionally, noise is unavoidable while gathering visual data because of the intricacy of the underwater world and illumination. The target missed rate is particularly high because underwater image difficulties frequently disrupt the underwater target detection network during training. Faced with these obstacles, conventional target detection technology’s depth has been insufficient to fulfill the demands of current industrial applications. As a result, there is an urgent need for an effective and lightweight target detection algorithm that may be deployed on compact, flexible detection devices. Using this compact equipment instead of huge exploratory equipment lowers detection costs and reduces human resource usage.

Previously, the target identification operation was primarily done by hand. This had the disadvantage of making the detection result susceptible to subjectivity and having a very sluggish pace, which was unable to fulfill the demands of real-time detection. For many years, researchers [4] have examined object detection tasks [5–7]. Prior to the development of deep learning technologies, object detection is usually accomplished through three stages: coordinate regression, feature extraction, and classification, followed by region suggestion. Typically, region suggestion generation creates a large number of potential boxes on the picture using sliding windows of varying sizes or selective search methods. SIFT, HOG, and other descriptors extract the fixed-length feature vectors based on these candidate boxes. Finally, SVM, artificial neural networks, and other classifiers are utilized to perform coordinate regression and classification prediction. Nevertheless, a lot of candidate boxes unrelated to the detection target will be generated by the sliding window’s region suggestion generation, wasting scarce computer resources in the target recognition process. These descriptions are also manually created. Underwater photos with inadequate lighting and imaging make it difficult to successfully detect the target characteristics, resulting in poor generalization ability and a substantial error between the findings and the real results.

Object detection has advanced significantly as a result of the deep learning field’s quick development and the use of deep convolutional neural networks (DCNN) in computer vision applications. At present, many studies have shown [8,9] that the method based on deep convolutional neural network is significantly superior to the traditional method based on specific features [10]. The object detection technology based on deep learning uses a convolutional neural network (CNN) to extract the feature information of the object and trains it through a large number of labeled data. The network model can then automatically learn the features of the target by fitting the training data. This deep learn-based technique [11,12] eliminates the need to manually design descriptors and has better generalization and robustness. Starting with AlexNet winning the Massive Vision Challenge (ILSVRC), DCNN-based technology has sparked the deep learning craze with its high accuracy. More and more DCNN-based models are used for underwater target detection.

Based on this background, deep learning-based object detection algorithms are broadly classified into two groups: one-stage and two-stage techniques. The target detection task is split into two steps in the two-stage algorithm, represented by R-CNN [13], Fast R-CNN [14], and Mask R-CNN [15]. This method has high detection accuracy but a very slow detection speed that is unable to meet real-time requirements. The one-step algorithm, represented by the YOLO [16–18] series and SSD [19], compared with the two-stage algorithm, avoids the stage of producing regional recommendations and may immediately output the position and category of the target. Therefore, the one-stage method can satisfy the demands of real-time detection applications and has a high detection speed. With the development of target detection technology, the two-stage target detection algorithm is gradually being abandoned because of its heavy computation and slow detection speed. The one-stage algorithm achieves a better balance between accuracy and speed, making the one-stage detector popular.

Although the underwater target identification approach based on DCNN has been quite successful [1], it has certain flaws, such as poor detecting speed and an excessively huge model. The universality of target detection is the primary emphasis of the current SOTA [20,21] paradigm. These models are characterized by a large number of parameters and high computational requirements. Even if they can be deployed on underwater equipment, they will take a lot of resources of underwater equipment, and can not meet the detection of underwater targets. Therefore, it is necessary to develop a more lightweight and high-precision real-time underwater target detector, which is also the driving force of this research.

Based on the aforementioned explanation, a lightweight underwater target detection network (SCR-Net) is presented in this study. The method not only demonstrates outstanding accuracy and stability but also achieves significant optimization in performance. Additionally, by simplifying the model parameters and calculation quantity, the processing efficiency is increased and the computing burden is significantly decreased. The primary contributions of this study may be summarized as the following major points:

1. A new lightweight spatial pyramid structure is designed to reduce computational consumption and improve the computational efficiency of the model. By employing local aggregation and global integration, the SPPE module effectively reduces computation requirements and accelerates the model’s inference speed.
2. A lightweight cross-scale feature fusion pyramid structure is created. The CFFP structure utilizes multiple 1×1 convolutions in the neck network, significantly reducing the number of parameters and computational requirements while enhancing computational performance.
3. A new lightweight feature extraction module RGE is designed. General convolution replaces the Bottleneck module, commonly used in C2f. To mitigate performance loss from removing bottlenecks, RepConv convolution is employed in one of the gradient flow branches. This design effectively reduces the computation and parameter count of the model, greatly improving the inference speed.
4. Based on the aforementioned advances, SCR-Net, a novel deep learning-based lightweight underwater target detection method, is proposed. The computation and parameter quantities of this method are reduced by 55.5% and 37.1%, respectively. SCR-Net has achieved the best detection mAP@0.5 of 83.2% and 61.5% on two open-source underwater target datasets, outperforming the state-of-the-art lightweight models.

Next, this article is organized as follows. Related works section introduces the relevant research work of underwater target detection. The proposed methods section describes the basic theory and the methods proposed in this paper in detail. Results and discussions section introduces experimental materials and procedures. The suggested SCR-Net algorithm’s inventiveness and resilience are confirmed by means of an examination of the experimental results. The conclusions section mainly summarizes the research content of this paper, and then expounds on the future research direction.

## 2 Related works

### 2.1 Underwater target detection algorithm

Neural networks had their roots in the 1940s when the functioning of neurons was explained by mathematical models. The convolutional neural network (CNN) is a special kind of neural network, which is mainly used to process image and video data. In the underwater setting, CNN can efficiently extract features from photos and resolve issues with manual feature extraction. Villon *et al*. [22] used CNN to quickly identify fish in coral reefs. Guo *et al*. [23] used the deep residual network to identify sea cucumbers, and the accuracy rate was up to 89.53%. According to CNN, object detection has greatly benefited from the application of deep learning techniques. Underwater target detection technology has advanced significantly as a result of the success of general target detection technology. Based on process, underwater target detection can also be separated into two groups: one-stage and two-stage.

In a two-stage approach, Li *et al*. [24] used Fast R-CNN to fish data sets in a particular underwater habitat. The experiment demonstrated that compared to a detector based on manual feature extraction, the detection accuracy and speed are significantly greater. Faster R-CNN was used in conjunction with a classification network by Mandal *et al*. [25] to automatically identify and categorize several fish species in underwater movies. Lin *et al*. [26] devised an augmentation approach known as RoIMix and employed it in a two-stage detector similar to Faster RCNN, which can combine recommendations of areas obtained from distinct pictures. The findings demonstrate that on the Pascal VOC and URPC datasets, RoIMix enhances the performance of region-based target detectors. Qi *et al*. [27] suggested a two-stage underwater small target detector with a deformable convolution pyramid (DCP) to address deformation, occlusion, and a variety of object sizes.

To address the two-stage detector’s detection speed issue, Sung *et al*. [28] created a target detection algorithm based on YOLOv1 for fish video recognition, achieving a classification accuracy of 93% and a detection speed of 16.7FPS. Compared to the two-stage detection approach, this detection speed is substantially greater. Liu *et al*. [29] coupled the YOLOv3 with the Domain Invariant Module and Invariant Risk Minimization penalty. Subsequently, a DG-YOLO model was suggested, achieving strong domain generalization performance in the identification of underwater targets. Zhao *et al*. [30] suggested a YOLOv4-based detection methodology for dead fish in aquaculture. This model substitutes deep separable convolution for regular convolution, with MobileNetV3 serving as the backbone network. The FPS doubles and the number of parameters is ten times lower than with YOLOv4. Aiming at the issues of tiny, noisy targets and dense targets in submerged settings, Xu *et al*. [31] included attention feature fusion and multi-scale feature extraction into the YOLOv5 algorithm. The mAP on the URPC2020 dataset has grown by 3.6% to 53.4% in comparison to the original YOLOv5. Zhang *et al*. [32] employed the modified YOLOX algorithm to detect marine animals, achieving an average accuracy of 70.9% across diverse types of marine species. Zhang *et al*. [33] created an effective lightweight convolutional neural network in response to the challenges associated with implementing deep convolutional neural networks on embedded devices. When fewer parameters and computations are used, the detection accuracy is similar to the SOTA model. Lv *et al*. [34] integrated the RT-DETR backbone into YOLOv8 and enhanced its capability for detecting underwater bridge cracks using PKI modules and context anchor attention. The model achieved an FPS of 87, enabling real-time underwater detection. Although these methods have had some success, given that they frequently demand additional processing resources, deploying underwater unmanned equipment remains a difficulty.

### 2.2 Lightweight network

In recent years, due to the continuous progress of technology, the accuracy and speed of target detection model have been greatly improved, and it is widely used. Because of different application scenarios, different detection models will be used. In some resource-constrained devices, such as drones, mobile robots, etc., the hardware is limited, resulting in a significant decline in the performance of the detection network, or it cannot be deployed. Therefore, lightweight object detection models need to be deployed on these devices to solve this problem.

Mainstream lightweight object detection models include MobileNetV3 [35], ShuffleNet [36], etc. They improve the performance of the network by updating the structure of the network and applying different convolution methods. Tan *et al*. [37] used the weighted BiFPN structure to integrate different network features to improve the efficiency of the network under resource constraints. Chen *et al*. [38] used new local convolution to reduce memory access and reduce model size to design a small network called FasterNet, which achieved low memory usage and high performance.Zhang *et al*. [39] designed a dual-path enhanced channel attention module based on YOLOX, optimized the loss function, and incorporated a receptive field module in the detection head. These improvements significantly reduced the parameters and floating-point operations of their proposed FDNet network. Chen *et al*. [40] designed a lightweight aggregation network (underwater-MLA) to reduce resource consumption in underwater devices. By integrating a multi-branch structure with hybrid convolution and contextual attention, along with the introduction of the Focaler-IoU module, the model significantly reduces complexity.

Another popular approach is to use lightweight detection networks as feature extraction modules in the backbone. For example, Wu *et al*. [41] replaced the feature extraction module in YOLOV5 by MobileNetV3 to reduce the network scale and improve the detection speed, so as to solve the situation of limited resources of unmanned devices. Guo *et al*. [42] used lightweight FasterNet to replace the backbone network in YOLOV8 to achieve faster and lighter underwater target detection. In addition, Cui *et al*. [43] capture global context features through a lightweight transformer block to enable real-time detection and model lightweight. Zhao *et al*. [44] introduced a novel lightweight detection model, FEB-YOLOv8. The lightweight and accuracy of the model are considered by adding the novel C2f and EMA modules and creating a new feature pyramid network topology. These are all efforts to enable devices to use object detection models in resource-constrained situations. The following is the work done in this paper for the resource limited device to be able to use the target detection model normally.

## 3 The proposed methods

**Fig 1:**
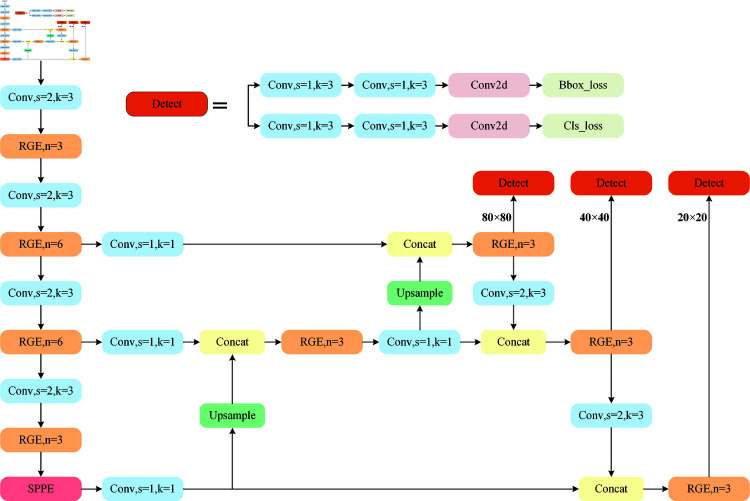
Structure of SCR-Net.

Fig 1 shows the structure of the proposed network model. The model as a whole realizes more comprehensive feature fusion through the top-down and bottom-up paths, while maintaining the efficiency and accuracy of the network. Contents of each chapter are arranged as follows: In Spatial Pyramid Pooling ELAN Section, the pyramid pool structure that is relevant to this research is introduced, and the construction procedure of SPPE is explained. The neck structure of the optimized network-CFFP, is presented in Cross-scale Feature Fusion Pyramid section. The suggested RGE module, which facilitates quicker feature extraction during inference, is introduced in Improved feature extraction module section.

### 3.1 Spatial pyramid pooling ELAN

The SPPE block proposed in this paper is based on Spatial Pyramid Pooling Fast (SPPF) and Efficient Layer Aggregation Networks (ELAN). Before building the modules, the basic information about SPPF and ELAN will be briefly introduced. We then integrate the SPPE we built into the end of the backbone network.

#### 3.1.1 Spatial pyramid pool structure

**Fig 2:**
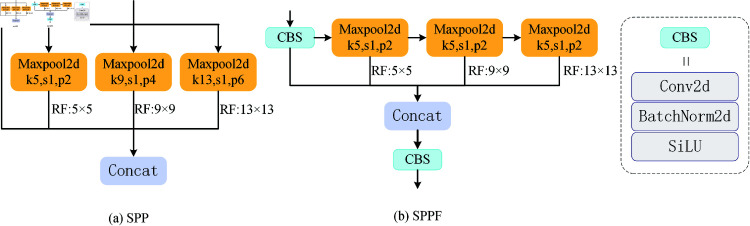
Structures of SPP and SPPF. (a) Spatial Pyramid Pool. (b) Spatial Pyramid Pool Fast.

Spatial Pyramid Pooling (SPP) is a spatial pyramid pooling strategy introduced in YOLOv3, which can capture spatial information at different scales to enhance the robustness of the model. However, the parallel structure in SPP increases the computational load and affects the inference speed of the model. In order to address this issue, YOLOv5 proposed SPPF, which concatenates three convolution nuclei of the same size in order to increase performance and decrease computation. Fig 2 depicts the SPP and SPPF structures. By splicing the pooled features with the original features and performing multi-scale pooling operations, the SPPF module may improve the expressive power of feature maps. The primary concept is to increase the model’s inference speed by minimizing computational redundancy while keeping the SPP function. In particular, SPPF reduces computational complexity by combining pooling processes that were originally carried out on numerous scales into a single scale using pointwise convolution and grouped convolution methods. Even in an underwater setting with constrained resources, SPPF retains an extensive amount of parameters and computational power. Thus, it is imperative to further optimize the SPPF structure to make it more appropriate for the underwater environment.

#### 3.1.2 Efficient layer aggregation networks

**Fig 3:**
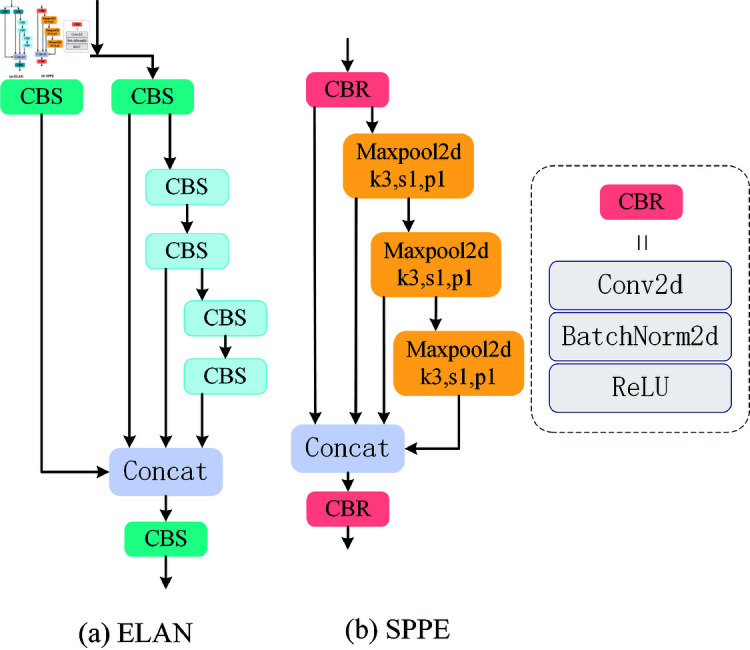
Structures of ELAN and SPPE. (a) Efficient Layer Aggregation Networks. (b) Spatial Pyramid Pooling ELAN.

ELAN in YOLOV7 [45] gathered more comprehensive gradient flow information by segmenting the gradient flow. By managing the shortest and longest gradient pathways, it allows the network to pick up more characteristics. To maximize the network’s gradient length, ELAN takes advantage of the stack structure of the module. ELAN is divided into two branches. The left branch uses 1×1 convolution to change the number of channels. The branch on the right employs four 3×3 convolution for feature extraction after adjusting the number of channels using 1×1 convolution. After a 1×1 convolution, the four features are finally concatenated and output. However, the additional joins and multiple convolutions introduced in ELAN still add quite a bit of computation. ELAN structures are shown in Fig 3a.

#### 3.1.3 The proposed SPPE

This research combines ELAN and SPPF to build the SPPE module, which can fully utilize ELAN’s effective feature aggregation ability and SPPF’s spatial pyramid pooling capacity to further enhance the model’s performance. The SPPE structure is shown in Fig 3b. The left branch of the ELAN structure is eliminated, and a maximum pooling layer is added instead of the first 3×3 convolution. The maximum pooling layer collects the most important characteristics in a given region, is appropriate for collecting edge and contour information, and is less computationally intensive. In contrast, 3×3 convolution will compute all features on an element-by-element basis, which requires a significant amount of computation and is unsuitable for resource-constrained applications. Furthermore, utilizing the maximum pooling layer can reduce network complexity and increase feature extraction efficiency.

Formula 1 illustrates how the original SPPF module uses the SiLU activation function. The SiLU function requires sigmoid function operation, which is extremely computationally intensive. ReLU functions require just one threshold operation, setting input values less than zero to zero. It does not need any sophisticated mathematical operations, which can effectively boost the model’s sparsity while reducing computing complexity. This makes ReLU better suited for application in contexts with high real-time demands and limited resources. In Formula 2, the ReLU function is displayed.

$$\text{S}iLU(\theta )=\frac{\theta }{1+e^{-\theta }}.$$ (1)

$$\text{R}eLU(x)=\max(0,w^{T}x+b).$$ (2)

### 3.2 Cross-scale feature fusion pyramid

**Fig 4:**
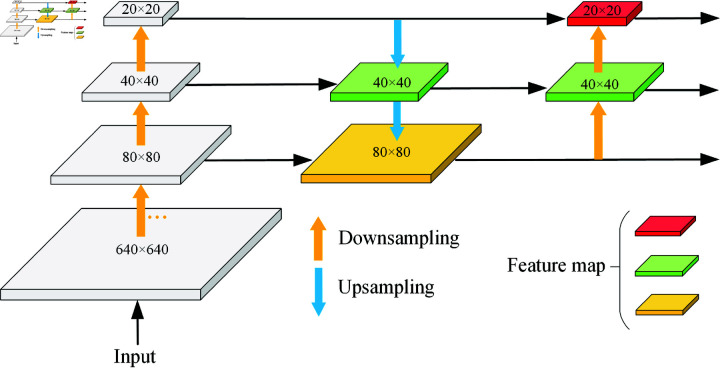
Cross-scale Feature Fusion Pyramid module.

Feature pyramid networks (FPN) are commonly employed in deep convolutional neural networks to fuse multi-scale features and resolve scale differences. To improve the semantic information of features, FPN presents a top-down network topology that blends high-level semantic information with low-level spatial information. On the basis of FPN, the path aggregation network (PAN) adds a bottom-up path aggregation network that may improve the feature details. PAN specifically enables the connection between feature maps sampled from high-resolution feature maps and feature maps sampled from low-resolution feature maps. The outcomes of combining various feature map layers are then cascaded. This can reduce information loss and retain more detailed information, thus improving detection accuracy. On this basis, the dual-stream PAN-FPN combines the benefits of both FPN and PAN and can fully utilize distinct levels of feature information to increase detection accuracy via top-down and bottom-up feature fusion. A large number of experiments proved that increasing the amount of processing within the feature pyramid solved the scale variance problem. However, the presence of several pathways results in a large rise in the computation and parameter number, as well as a considerable increase in the complexity of the network structure. Any extra calculation will have an effect on equipment used in an underwater environment. As a result, cutting out pointless computations is crucial. In order to decrease the computational load, this article improves the feature pyramid network by adding multiple 1×1 convolutions in comparison to the standard PANet. Fig 4 illustrates the suggested CFFP design.

**Fig 5:**
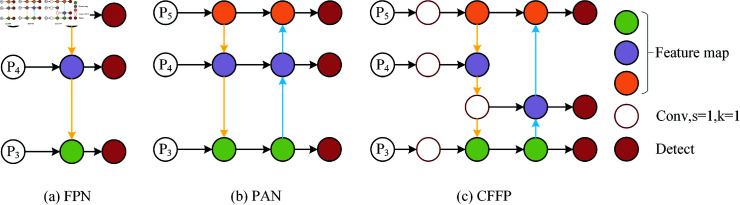
Three different feature pyramid structures. (a) A classic multi-scale feature fusion network with a top-down path. (b) A bottom-up path is built on top of (a). (c) A cross-scale feature fusion pyramid.

In general, the feature maps of the three backbone scales are spliced after up-down sampling to improve the network’s feature representation capabilities. This paper designs a cross-scale feature fusion pyramid structure. Three 1×1 convolution operations are added between the neck network and backbone network to keep the number of output channels of the feature graphs of different sizes at 64, thereby reducing the number of calculations and parameters. This strategy can greatly lower the computational cost of future feature fusion. The quality of the feature map is improved by 1x1 convolution, which reweights and modifies the input feature map via linear transformation, enhancing significant characteristics and suppressing unimportant ones. Fig 5 displays the unique CFFP structural diagram.

Furthermore, we incorporate a 1x1 convolution into the neck network. Following feature fusion, the feature map includes features from many layers that differ somewhat in terms of information, orientation, and scale. Before upsampling, 1 × 1 convolution can realign and fuse these features, guaranteeing their consistency and adaptability. While there is no change in the number of channels at this point, the inclusion of 1 × 1 convolution offers a simple renormalization and correction of the feature graph without adding to the computational load.

### 3.3 Improved feature extraction module

#### 3.3.1 Reparameterization and RepConv

**Fig 6:**
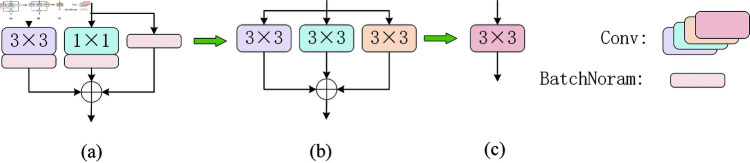
Structure of RepVGG block.

Reparameterization technique is a technique used in deep learning to optimize model performance and inference efficiency. Its main idea is to use complex multi-branch structures during the training phase to enhance the model’s expressive ability, and then simplify these multi-branch structures into a single convolutional structure during inference to reduce computational complexity and memory consumption. The reparameterization technique was applied in the RepVGG [46] network, and its basic principle is shown in Fig 6. During training, the structure shown in Fig 6a is used. However, during inference, all operations are simplified to a simple 3×3 convolution as shown in Fig 6c. This technique not only greatly simplifies the original complex structure but also does not reduce the model’s performance.

RepConv is a convolutional neural network module that addresses the high computational complexity of convolution operations in traditional neural networks. RepConv enhances the model’s feature extraction ability through the use of reparameterization techniques. The core idea behind RepConv is to decouple the weights and biases in traditional convolutional layers, thereby improving the network’s expressive ability and flexibility. RepConv uses a multi-branch convolution layer in training, and re-parameterizes the parameters of the branches to the main branches in inference, thus reducing the computation and memory consumption, which is very suitable for resource-limited environments.

#### 3.3.2 The proposed RGE

Kai Han *et al*. [47] proposed GhostNet, a lightweight convolutional neural network architecture, in 2020. This research mentions that while computing intermediate feature mappings, classic convolutional neural networks include a lot of redundancy. To do this, Kai Han *et al*. presented a novel technique for creating feature maps in GhostNet, which involves creating duplicate feature maps via inexpensive operations, greatly lowering the number of parameters and processing required. In addition, ELAN structures can obtain detailed information by segmenting the gradient flow. When extracting features, the application of ELAN structure can greatly increase the ability to extract detailed features.

**Fig 7:**
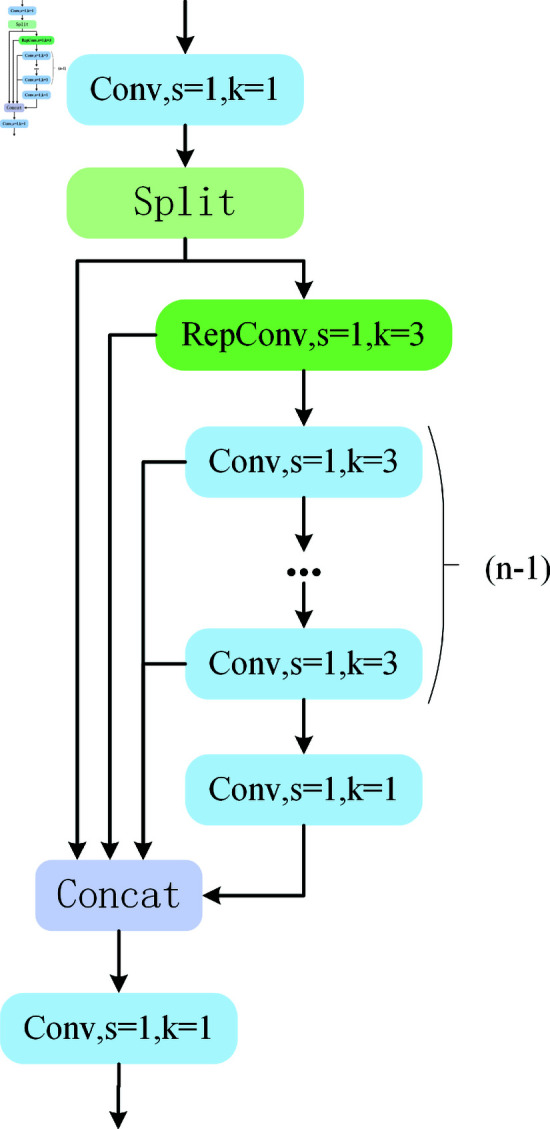
Structure of RGE.

On this premise, RGE, a novel lightweight feature extraction module, is presented. Its structure is shown in Fig 7. The widely used BottleNeck module in C2f is discontinued by the RGE module. We employ RepConv convolution on gradient flow branches to improve gradient flow and feature extraction capabilities, making up for the little performance loss resulting from the removal of residual blocks. Furthermore, the original complicated multi-branch structure is reduced to a single convolution operation in the inference phase using RepConv’s reparameterization technique, which significantly lowers the compute and memory consumption. In the Fig 7 (*n*–1) shows how many 3×3 convolutions there are.

In the RGE module, the size of the RGE may be modified by the scale factor value, allowing it to accommodate both tiny and big models. We used the scale value of 0.5 to find a compromise between feature representation, computational efficiency, and practical application needs. Scale specifically determines the amount of intermediate feature channels. Setting it to 0.5 can minimize the model’s parameter count and computational cost while retaining enough feature representation capacity, enhancing the model’s overall operating efficiency. This decision is based on best practices for lightweight models like MobileNet and ShuffleNet, and similar scale values work well in a variety of use scenarios. Furthermore, scale=0.5 not only assures that the module is lightweight, but it also boosts its versatility in various workloads and deployment scenarios.

## 4 Results and discussions

This part evaluates the suggested model and a series of approaches using a large number of experiments and performs the related analysis. To be more specific, the training details, data sets, experimental assessment indications, and experimental environment are presented first. After starting from scratch and training the model on the DUO dataset, ablation tests were conducted to confirm the efficiency and performance of RGE, SPPE, and CFFP. Simultaneously, we assess the suggested model’s performance on the DUO and UDD datasets against the most advanced lightweight frameworks available today. To confirm that the suggested model functions as intended, the findings are examined and condensed. Furthermore, each model’s consequences in applications related to underwater environments are also visualized.

### 4.1 Material details

#### 4.1.1 Experimental environment

Using data sets, the model described in this study is trained and evaluated on a general-purpose computer. Table 1 displays the machine’s exact setup.

**Table 1:** Detailed specifications of the computer.

Parameter	Configuration
CPU	Intel(R) Core(TM) i9-11900K
GPU	NVIDIA GeForce GTX 3090 (24GB)
Operating system	Windows 11
torch	2.0.1
Python	3.8.17
CUDA	11.8
cuDNN	8.7.0
Pre-trained weight	No
Batch size	32
Input size	640×640 pixels
Optimizer	SGD
Learning rate	0.01
Weight decay	0.0005
Momentum	0.937
Epochs	400

#### 4.1.2 Evaluation metrics

The model presented in this work is assessed using the recall rate, computational correctness, and confusion matrix displayed in Table 2. Formulas 3 and 4, respectively, can be used to represent the precision and recall values. A correct defect prediction is denoted by TP, an inaccurate defect prediction by FP, an incorrect background prediction by FN, and an accurate background prediction by TN.

**Table 2:** Confusion matrix.

Label	Predict	Confusion matrix
Positive	Positive	TP
Positive	Negative	FN
Negative	Positive	FP
Negative	Negative	TN

$$Presion=\frac{TP}{TP+FP}.$$ (3)

$$Recall=\frac{TP}{TP+FN}.$$ (4)

However, accuracy and recall ratings only measure a portion of the model’s performance. The most essential and often used assessment metric in object identification algorithms is mean Average Precision (mAP). The Precision-Recall Curve was produced using the recall rate as the horizontal axis and the accuracy rate as the vertical axis. Average Precision (AP) is the area under the PR curve. In general, the better the classifier, the higher the AP score. The calculation formula for AP is Formula 5, where P represents Precision and R represents Recall rate. The mAP is the average of APs, and k is the number of classes, as shown in Formula 6.

$$AP=\int_{0}^{1}P(R)dR,$$ (5)

$$mAP=\frac{1}{k}\sum_{i=1}^{k}AP_{i}.$$ (6)

The model’s size and complexity are indicated by the total number of parameters (Params) and FLOPs. For instance, the convolutional layer’s parameters are determined using Formula 7:

$$Params_{conv}=C_{out}\times (K_{w}\times K_{h}\times C_{in}+1),$$ (7)

where the width and height of the convolution kernel are denoted by *K*_*w*_ and *K*_*h*_ in the formula, respectively, while the number of input and output channels is represented by *C*_*in*_ and *C*_*out*_.

Formula 8 displays the convolution layer’s FLOPs mode:

$$FLOPs_{conv}=F_{h}\times F_{w}\times \left(C_{in}\times \frac{2k^{2}-1}{g}+1\right)\times C_{out},$$ (8)

where g is the convolution’s group size, *k* is the convolution kernel’s size, and *F*_*h*_ and *F*_*w*_ are the input feature map’s width and height, respectively. MegaParams (M) are the standard unit of measurement for parameters in target detection models, while gigaFLOPS (G) are the standard unit of measurement for FLOPs.

Image preparation, inference, and non-maximum suppression (NMS) are steps in the image detection process. Thus, Formula 9 can be used to define the speed of the model as the average time it takes to detect all pictures.

$$\text{Speed}=\frac{\sum_{i=1}^{n}t_{i}}{n},$$ (9)

where *t*_*i*_ is the total amount of time needed to process the *i*-th picture, and *n* is the total number of photos processed.

### 4.2 Dataset preparation

Two difficult and open-source datasets, DUO and UDD, are selected for the experiment. A recently published underwater dataset called DUO [48] has more precise categorization and covers many different kinds of underwater environments. As an improved version of the UTDAC2020 dataset, DUO inherits the rich qualities of the URPC2020 Underwater Target Detection Challenge, while also improving data quality and structure. There are 7,782 photos in all, divided into four popular categories: scallops, sea cucumbers, starfish, and sea urchins. There are four resolutions for the photos in the dataset: 1920 × 1080, 720 × 405, 586 × 480, and 3840 × 2160.

The Dalian University of Technology underwater robot team produced the UDD [49] dataset, an underwater image dataset, for the robot target acquisition job. With a total of 2227 photos, the collection is divided into three categories: sea urchins, sea cucumbers, and scallops.

The DUO and UDD datasets are pre-processed by first converting them to VOC format, and then converting them back to their original forms based on the requirements of each comparison experimental model. We utilize YOLOv8’s default normalization (scaling pixel values to the range of [0,1]) and common data augmentation techniques, such as random cropping, horizontal flipping, and color jittering, to simulate underwater scenarios with varying lighting conditions and random target poses, thereby enhancing the model’s generalization ability and robustness. We divided the pre-processed data set and ensured the picture and label partitions were consistent for every model format to guarantee the correctness of the experiment. The DUO and UDD datasets were split into training, validation, and test sets at random for this investigation, with the ratios being 0.70:0.15:0.15. During training, the picture size was uniformly adjusted to 640×640.

### 4.3 Ablation experiments and results visualization

#### 4.3.1 Ablation studies for SPPE

We performed ablation experiments on the baseline model, using the default configuration of the model. Through a performance comparison with the current spatial pyramid pool module, SPPE’s good performance and lightweight construction are confirmed. Table 3 displays the outcomes of all the tests that were performed using the DUO dataset. SPPE has the best overall performance.

**Table 3:** Experimental comparison of different spatial pyramid pooling structures.

Models	Params/M	FLOPs/G	mAP@0.5/%	Speed/ms
SPP	3.01	8.1	82.9	9.3
SPPF	3.01	8.1	82.8	9.2
SimSPPF	3.01	8.1	83.4	9.7
ASPP	5.07	9.8	82.5	9.5
SPPE	2.85	8.0	83.4	9.1

Based on the DUO dataset, the experimental findings indicate that there is minimal computational difference between SPP, SPPF, and SimSPPF. SimSPPF has greater detection performance, but its inference speed is the worst of any module. The mAP of SPPE is the same as that of SimSPPF, but it has an inference speed of only 9.1 ms and fewer parameters—a reduction of 5.3% was achieved. This demonstrates that it is lighter and more accurate to use ELAN’s efficient polymerization capabilities and lightweight features rather than SPPF structures.

#### 4.3.2 Ablation studies for all

To validate the increased performance reported in this study, we perform ablation experiments on the DUO dataset with changes to SPPELAN, CFFP, and RGCE. The first YOLOv8n model serves as the baseline. Experiments with ablation were conducted on the subsequent network: S stands for the SPPE module replacing the SPPF module in the baseline model. R stands for replacing every C2f module with an RGCE module in the baseline model. C represents the CFFP structure designed in this paper in the neck structure of the model.

We examined 1168 images to verify the performance of SCR-Net. The ablation experiment results, including the number of parameters, calculation amount, mAP@0.5, and Speed, are shown in Table 4. As can be seen, YOLOv8n has achieved good results on the publicly available DUO dataset. The improvements proposed in this paper will then push the metrics to get better and make the model more lightweight.

**Table 4:** Ablative experiment results.

Models	Params/M	FLOPs/G	mAP@0.5/%	Speed/ms
YOLOv8n	3.01	8.1	82.8	9.2
+S	2.85	8.0	83.4	9.1
+R	2.23	6.2	82.6	8.9
+C	2.07	7.3	83.5	9.5
+C +S	1.81	6.6	82.9	9.4
+C +R	1.50	5.2	82.7	9.3
+C +R +S	1.34(-57.4%)	5.1(-37.0%)	83.2	9.3

Initially, we conducted tests on the three modules to investigate how each affected the model’s overall performance. Substituting the previous SPPF with the new SPPE results in a 5.3% reduction in parameters and 0.1G less floating-point calculations in the model when compared to the baseline. When compared to the baseline model, there is a 0.6% improvement in average accuracy. A single image’s reasoning speed of 9.1 ms was achieved, indicating a marginal increase over the baseline model and further proof that the lightweight nature of ELAN accelerates the reasoning speed of the model. When all of the C2f modules in the baseline model were replaced with the RGCE module, the number of model parameters and floating-point computations were decreased by 25.9% and 1.9G, respectively. The testing findings show that the model reaches 8.9 ms reasoning speed by using the re-parameterization approach to reduce the module’s structure, which requires less time than any other model. The number of parameters is lowered by 31.9% when the CFFP structure is utilized exclusively. There is a 0.7% improvement in the model mAP@0.5’s average accuracy. This further demonstrates the dependability of our addition to the neck network of 1×1 convolution processes. Even with the little inference time delay that has been caused by the CFFP structure, the inference speed is still fast enough to satisfy real-time detection needs.

Next, we conduct tests based on the CFFP structure. The number of parameters and floating-point computations in the model are further decreased when SPPE alone is utilized in place of SPPF. Reasoning accelerated a little bit. The number of parameters and computation cost of the model is greatly decreased for each single module substitution when we substitute simply RGCE for all of the C2f modules. At this point, the model’s single picture inference speed was still 9.3 ms.

Lastly, there are clear benefits of the SCR-Net shown in this study above the baseline model. SCR-Net had 1.34 M parameters and 5.1 G of computation, which were respectively 55.5% and 3.0 gigabytes (37%) less than the baseline model. mAP@0.5 was 0.4% greater than the baseline model, reaching 83.2%. A single image’s inference speed may reach 9.3 ms, which is fast enough to identify underwater pictures in real-time.

**Fig 8:**
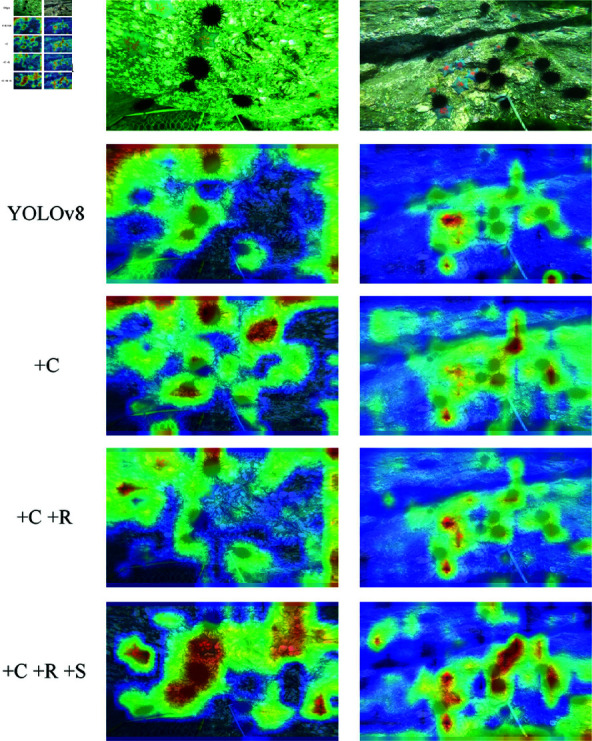
Heat maps produced by different methods.

To further illustrate the various modules’ contributions to the entire model, we created heat maps using two randomly chosen photos from the test set, as seen in Fig 8. Sea urchins and starfish are the two kinds of targets seen in both images. The first image’s color is darker, making it difficult to distinguish the target’s color from the backdrop. The starfish on the left in the second picture is in the center of the cracks and has a dark backdrop. In both images, the starfish features are poor and are affected by lighting and shooting angles.

Fig 8 illustrates how the cross-scale feature fusion pyramid construction makes the target characteristics more visible. The features that RGCE can extract are sufficient for detection. Following the integration of the three techniques into SCR-Net, the feature map exhibits enhanced color richness and improved target detection accuracy in terms of location. Moreover, SCR-Net is efficient in concentrating attention on elusive things, such as the starfish situated on the left side of the second picture. Overall, the model’s performance can be successfully enhanced in the three suggested ways, and this heat map aligns with the ablation experiment’s description.

**Fig 9:**
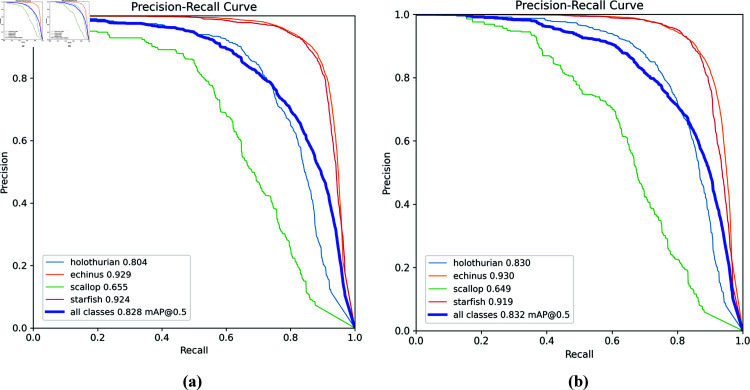
Evaluation of performance of YOLOV8n model and SCR-Net model. (a) YOLOV8n. (b) SCR-Net.

Furthermore, we compared the performance of the SCR-Net model we created to the YOLOV8n model on the DUO dataset, as shown in Fig 9. Interestingly, AP rose by 2.6% in the "holothurian" category. The mAP value improved by 0.4% over the baseline model to 83.2%. These improvements demonstrate that the SCR-Net model outperforms the original model.

#### 4.3.3 Ablation studies for backbone

To assess the influence of various lightweight backbone networks on model performance, MobileNetV3 [50], EfficientNet [51], ShuffleNetv2 [52], and GhostNetv2 [53] were employed as backbone networks based on the baseline network (YOLOv8 [54]) and compared to SCR-Net. Table 5 summarizes the experimental outcomes. It should be emphasized that we did not change the backbone directly in SCR-Net, but rather tested it by replacing the backbone in the baseline network. The experimental results validate SCR-Net’s robustness and effectiveness in handling complex underwater images. Despite variations in efficiency and performance across different lightweight backbone networks, SCR-Net consistently outperforms them under various underwater image degradation conditions. These experimental results highlight SCR-Net’s superiority over other lightweight detectors.

**Table 5:** Comparison of different backbone networks on DUO datasets.

Backbone	Params/M	FLOPs/G	mAP@0.5/%	Speed/ms
YOLOv8 [54]	3.01	8.1	82.8	9.2
MobileNetV3 [50]	2.35	5.4	77.4	12.4
EfficientNet [51]	1.90	5.6	80.2	11.9
ShuffleNetv2 [52]	1.71	5.0	74.0	10.1
GhostNetv2 [53]	3.78	6.7	80.0	21.9
SCR-Net	1.34	5.1	83.2	9.3

#### 4.3.4 CFFP features enhanced visualization

**Fig 10:**
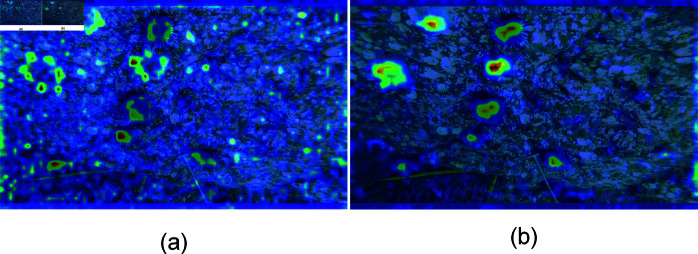
CFFP features enhanced visualization (a) feature map before CFFP. (b) feature map after CFFP.

Fig 10 depicts the activation of the feature map before and after passing through the CFFP structure. In the comparison of thermal maps in Fig 10a (before CFFP) and Fig 10b (after CFFP), the CFFP module has a considerable influence after feature fusion. In Fig 10b, the activation intensity of the target region is greatly increased, while the attention of the target-independent region is significantly decreased, suggesting that the CFFP module efficiently reduces information loss through multi-scale feature fusion and channel optimization. The activation diagram indicates that following CFFP, the target region’s features are more focused, background interference is minimized, and target features become more prominent. This demonstrates that the CFFP module not only improves the representation of target information, but also increases attention to the target, hence increasing the model’s detection performance.

#### 4.3.5 Visualization of robustness to noise and degradation

**Fig 11:**
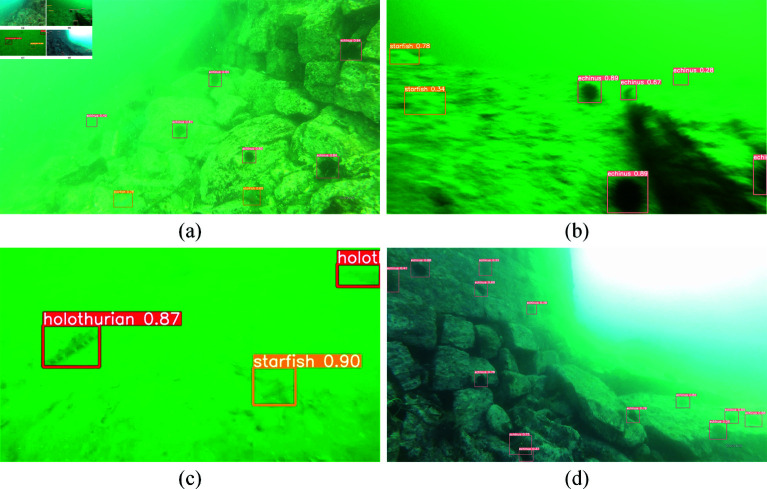
Visualization of noise and degradation robustness. (a) hazing. (b) blur. (c) low contrast. (d) imbalanced light conditions.

To further evaluate the performance of SCR-Net under degradation conditions common in underwater environments, such as noise, blur, and color distortion, we conducted experiments and presented the results in Fig 11. Specifically, the trials emulated various degradation conditions, such as adding noise, introducing blur, and simulating color distortion caused by variations in underwater light and depth. The model’s detection effect under these degradation conditions is depicted in Fig 11. The experimental results show that, even with typical underwater image deterioration, SCR-Net can retain steady detection performance and durability. This suggests that SCR-Net has significant utility and potential in complex underwater application settings.

### 4.4 Comparison to state-of-the-art models

#### 4.4.1 Experimental results on the DUO dataset

Using DUO optical pictures, we investigate and evaluate a few popular and current lightweight object detection methods to confirm the robustness of the paradigm provided in this research. FastestDet [55], YOLOv3-Tiny [18], YOLOv5n [56], YOLOv5s [56], YOLOv7-Tiny [57], YOLOv8n [54], and YOLOv10n [58] are among the techniques that fall under this category. The comparative experiment uses the same data sets and divisions as the previous one to ensure fairness. Table 6 displays the outcomes of the experiment.

**Table 6:** Comparison results of different models on the DUO dataset.

Models	Params/M	FLOPs/G	mAP@0.5/%	Speed/ms
FastestDet [55]	0.23	0.42	62.3	14.8
YOLOv3-Tiny [18]	8.67	13.0	74.7	13.5
YOLOv5n [56]	1.90	4.5	81.7	12.8
YOLOv5s [56]	7.07	16.5	82.0	13.6
YOLOv7-Tiny [57]	6.02	13.2	77.4	14.0
YOLOv8n [54]	3.01	8.1	82.8	9.2
YOLOv10n [58]	2.71	8.4	82.7	11.8
Ours	1.34	5.1	83.2	9.3

On the DUO dataset, the model proposed in this research performs better than the sum of the performance of these comparator models. The model’s mAP@0.5 stands at 83.2%, and its reasoning speed for a single picture is a mere 9.3 ms. In terms of the number of parameters and the amount of effort needed, the FastestDet is extremely lightweight, but it also loses speed and precision.

**Fig 12:**
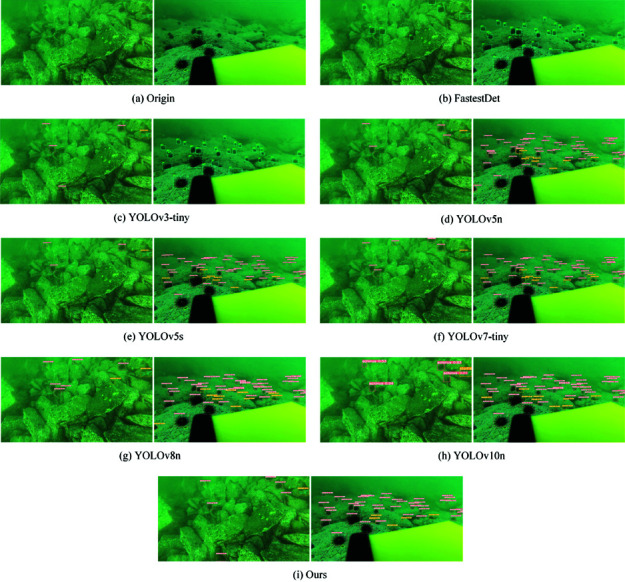
Visualization of test results for different models on DUO datasets.

In this work, visualization experiments have also been performed to demonstrate the efficiency of the suggested model. We used the model shown in Table 6 above and chose two test photos at random to compare. Fig 12 displays the results of the visualization. The categories and confidence levels of the objects present in every region of the test picture are represented by the anchor box. The model presented in this work is also able to detect well for the target that is detectable by all the models in the image. Two nonexistent items are incorrectly recognized by the YOLOv8n model in the center of the first shot. In the image’s top right corner, YOLOv8n missed an apparent target. This paper’s model does a great job of avoiding these two mistakes. Regarding the second graph, the model presented in this research is more confident in detecting clear targets and is also capable of detecting fuzzy objects that are far away. In conclusion, the model in this study has a higher detection rate, can identify targets that other models are unable to detect, and has also attained SOTA model detection accuracy. As a result, the model presented in this work produces superior outcomes.

#### 4.4.2 Experimental results on the UDD dataset

In addition to testing on the DUO dataset, we also tested on the UDD underwater dataset to confirm the universality of the model provided in this study. The experimental findings are displayed in Table 7 and the model utilized in the experiment is the same as the one employed in the test on the DUO dataset.

**Table 7:** Comparison results of different models on the UDD dataset.

Models	Params/M	FLOPs/G	mAP@0.5/%	Speed/ms
FastestDet [55]	0.23	0.42	45.3	22.9
YOLOv3-Tiny [18]	8.67	13.0	53.5	8.4
YOLOv5n [56]	1.90	4.5	59.2	11.0
YOLOv5s [56]	7.07	16.5	59.9	11.7
YOLOv7-Tiny [57]	6.02	13.2	44.8	12.6
YOLOv8n [54]	3.01	8.1	60.3	10.4
YOLOv10n [58]	2.71	8.4	58.0	11.8
Ours	1.34	5.1	61.5	10.0

**Fig 13:**
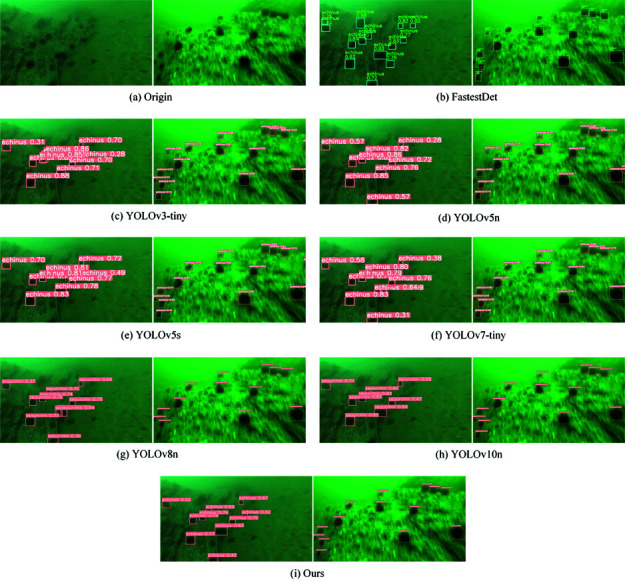
Visualization of test results for different models on UDD datasets.

The experimental findings shown in Table 7 demonstrate that the model used in this work continues to have the highest overall performance. With an average accuracy of 61.5%, 1.2% higher than the baseline model, it is the highest accuracy of all the models. A single image’s inference time is 10.0 ms, which is only slower than the YOLOv3-Tiny model. Two randomly chosen photos from the test set were also used for testing with the UDD data set; the visualized images are displayed in Fig 13. The article model in the first figure can identify every target that the other models have identified. A minor target in the middle of the image was overlooked by the baseline model, and our model recognized most objects with a higher confidence level than the baseline model. Regarding the second image, our model remains successful in detecting tiny targets and occluded targets even in cases when the image is hazy. The model proposed in this paper has a strong detecting capability.

### 4.5 Failure case analysis

While SCR-Net demonstrated superior performance compared to state-of-the-art lightweight models, certain failure cases were observed under challenging underwater conditions. Specifically, the model exhibited susceptibility to false positives in highly occluded or low-contrast environments. For instance, during experiments on the DUO dataset, when sea urchins were partially obscured within complex underwater terrains, SCR-Net occasionally misclassified background textures as marine organisms. Similarly, in low-light scenarios or turbid water conditions, the model sometimes failed to distinguish actual marine organisms from image noise artifacts, resulting in incorrect detections.

Another notable issue emerged when multiple objects were closely clustered together. In certain instances, the model struggled to differentiate individual instances, either merging them into a single detection or producing multiple overlapping bounding boxes for a single object. This indicated that, while the cross-scale feature fusion mechanism improved detection accuracy, further refinement might be required to enhance object separation in dense environments.

These failure cases suggest that SCR-Net, though effective in most scenarios, encountered limitations under extreme environmental conditions. A detailed analysis of these challenges could offer valuable insights for enhancing the model’s robustness in future iterations.

## 5 Conclusions

This study develops a deep convolutional neural network that is accurate and lightweight, to lower the resource consumption of underwater equipment that has been used for target recognition. The present issues of huge model size, large computation, and high parameter quantity are resolved by the SCR-Net proposed in this research. The model has been improved in several ways. First, a brand-new, SPPE is intended for the backbone network’s terminus. It can efficiently lower the model’s number of parameters and computation load, and it also quickens the network’s rate of reasoning. Furthermore, a new feature extraction module called RGE is suggested. This module may produce redundant feature maps using inexpensive operations, significantly lowering the model’s parameter count and computation costs. The reparameterization technology used by the RGE module combines several computer modules into one, significantly reduces the amount of time required for inference. Lastly, the feature fusion layer’s structure is altered. The input feature fusion layer’s channel number of 64 is achieved by a series of simple convolution processes. The model’s computational redundancy is significantly decreased by this approach. Following the fusing of feature maps with varying sizes, 1×1 convolution is then employed to minimize the small discrepancies and enhance the underwater target identification capability.

The ablation tests conducted on the DUO dataset have validated the efficacy of the suggested methodology. Furthermore, an extensive series of comparative studies on the DUO and UDD datasets, including a comparison with some of the most advanced lightweight models, confirmed that the suggested model performed optimally overall. The outcomes demonstrate that SCR-Net can achieve greater accuracy in real-time detection with less computational resources.

Although this study has made significant progress in lightweight underwater object detection algorithms, obtaining high-quality underwater image datasets remains challenging due to the complexity of the underwater environment. Factors such as lighting variation, water turbidity, and object motion further complicate data collection. These issues also lead to considerable differences between sample labels, affecting training performance. Therefore, future research will focus on exploring how image processing techniques can enhance underwater object detection performance, particularly under various underwater environmental conditions. In addition, the current method still has limitations in recognizing certain specific types of targets. Future work will validate the effectiveness of this approach on a broader range of underwater objects and further test the adaptability of SCR-Net in different scenarios. Furthermore, exploring methods to optimize SCR-Net for deployment on resource-constrained devices, such as underwater drones, will be an important direction for future work, ensuring the practical applicability of the proposed model in real-world underwater scenarios.
